# An ancestral glutamate receptor mediates cell volume regulation during high K^+^ stress in cyanobacteria

**DOI:** 10.1016/j.jbc.2026.111396

**Published:** 2026-03-21

**Authors:** Haoyu Zhang, Masaru Tsujii, Ellen Tanudjaja, Haruto Shimizukawa, Yuki Muraoka, Yuki Sato, Kota Kera, Tadaomi Furuta, Shingo Kaneko, Satoshi Amaya, Hirotaka Sugiura, Fumihito Arai, Yasuhiro Ishimaru, Nobuyuki Uozumi

**Affiliations:** 1 Department of Biomolecular Engineering, Graduate School of Engineering, Tohoku University, Sendai, Japan; 2 School of Life Science and Technology, Institute of Science Tokyo, Yokohama, Japan; 3 Graduate school of Engineering, The University of Tokyo, Bunkyo-ku, Tokyo, Japan

**Keywords:** *Synechocystis*, microfluidics, K^+^ channel, osmotic stress, glutamic receptor

## Abstract

Cyanobacteria, able to survive photoautotrophically in harsh environments, possess various ancestral homologs of ion transport systems found in eukaryotic cells. The model cyanobacterium, *Synechocystis* sp. PCC 6803, contains five K^+^ channels; however, their function and role have not been fully explained. This study determined the structure, function, and physiological role of an ancestral glutamate receptor, GluR0, in *Synechocystis*. Growth of a *gluR0* mutant (Δ*gluR0*) increased under high KCl conditions. Subcellular fractionation showed that GluR0 was localized in the plasma membrane of *Synechocystis*, and expression of GluR0 enabled a K^+^ uptake–deficient *Escherichia coli* mutant to grow under low K^+^ conditions. The membrane topology of GluR0 was opposite to that of the canonical K^+^ channel but similar to that of the animal glutamate receptor. Microfluidic device–aided single-cell analysis that enabled instantaneous extracellular solution exchange revealed that after KCl upshock, the cell volume of Δ*gluR0* decreased more rapidly than the wildtype. These data provide the first direct evidence that a prokaryotic glutamate receptor homolog with K^+^ channel activity plays a role in responding to rapid changes in the ionic environment. This function likely reflects a property of glutamate receptors that was acquired early on during evolution.

Microorganisms are equipped with molecular mechanisms to respond to rapid changes in their environment (1). Various ion transport systems in biological membranes function to regulate the formation of the membrane potential and maintain intracellular homeostasis in response to high osmotic pressure and high ionic strength (2). In both prokaryotic and eukaryotic cells, potassium ions (K^+^) are the main intracellular cations that significantly contribute to the generation of turgor pressure and membrane potential formation. K^+^ channels are present in nearly all cellular membranes, and they play a crucial role in the initial response to external changes (3).

Cyanobacteria are an ancient group of bacteria and are believed to have developed oxygenic photosynthesis early in evolution. This ability was later transferred to eukaryotic cells through the endosymbiosis of an ancestral cyanobacterium, leading to the emergence of algae and plants (4). *Synechocystis* sp. PCC 6803 was the first cyanobacterium whose genome sequence was completed, and it has been extensively studied as a model cyanobacterium (5). Cyanobacteria adapt well to different environments because of their great variety of membrane transporters. *Synechocystis* has one set of Trk/Ktr/HKT-type K^+^ transporters, KtrABE, and one set of Kdp-type K^+^ transporters, KdpABGCD (6). KtrABE serves as an Na^+^-dependent K^+^ uptake system that allows cells to recover from shrinkage because of high osmotic stress (6, 7, 8, 9), whereas Kdp acts as a high-affinity K^+^ uptake system, which plays a minor role in osmotic adaptation (6). The genome of *Synechocystis* contains at least five K^+^ channel homologs, more than that of *Escherichia coli*, which contains only one nonfunctional K^+^ channel homolog (10). SynCaK (*sll0993*) functions as a Ca^2+^-dependent K^+^ channel at the plasma membrane (PM) to confer resistance to Zn (11). SynK (*slr0498*) is a voltage-dependent K^+^ channel expressed in the plasma and thylakoid membranes (TMs) (12, 13, 14). SynK is involved in heavy metal resistance and promotion of photosynthetic activity of PSII. KirBac6.1 (*slr5078*) complements the growth of a K^+^ uptake–defective *E. coli* strain at low concentrations of K^+^ (15). GluR0 (*slr1257*) is a glutamate receptor homolog with K^+^ channel activity (16). Its three-dimensional structure has been solved, and binding of glutamic acid has been demonstrated (17). No functional information is available for the fifth K^+^ channel homolog, Sll0536.

Rapid volume changes occur in bacteria in response to changes in the external osmotic pressure and ion concentration. Cell volume changes because of rapid osmotic changes can be analyzed by measuring light scattering at 575 nm with a stopped-flow device (18). However, this analysis can only compare average values for cells in a suspension and is less accurate because changes in size because of the stress cannot be determined for individual cells. As an alternative approach, microfluidic devices can measure the volume change of a single *Synechocystis* cell during hyperosmolarity stress (6). More recently, development of an on-chip method has made it possible to more rapidly replace the external medium without damaging the cells (19). With this new technology, solutions with different osmotic pressures can be mixed in 3.6 ms, which greatly improves the time resolution of observing the response of single cells to osmotic changes. This development will further advance the analysis of cellular adaptation mechanisms in cyanobacteria.

In this study, we tested the growth of individual *Synechocystis* mutants for all five types of K^+^ channels in high concentrations of KCl. Based on our results, we then chose GluR0 for further analysis of its membrane topology and physiological properties. This is the first report demonstrating that a K^+^ channel is involved in the immediate response of cells to an increase in extracellular KCl concentrations.

## Results

### Involvement of GluR0 in adaptation to high KCl stress

**Figure 1:**
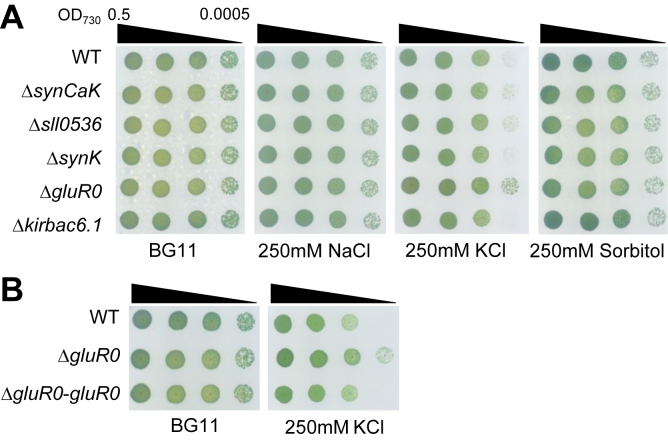
**Growth of the *Synechocystis* WT and five K^+^ channel mutants in medium containing 250 mM NaCl, KCl, or sorbitol.***A*, growth of the WT, Δ*synCaK*, Δ*sll0536*, Δ*synK*, Δ*gluR0*, and Δ*kirBac6.1*. Five microliters of cell suspension at an absorbance of 0.5 at 730 nm, followed by 10-fold serial dilutions, were spotted on BG11 medium with or without 250 mM NaCl, 250 mM KCl, or 250 mM sorbitol. Data shown are representative of three biological replicates. *B*, complementation of Δ*gluR0* by reintroduction of *gluR0*. Cells were spotted onto BG11 medium with or without 250 mM KCl, as in (*A*). Data shown are representative of three biological replicates.

Five genes encoding K^+^ channel homologs, *sll0993* for SynCaK (20), *sll0536* and *slr0498* for SynK (12, 13), *slr1257* for GluR0 (17), and *slr5078* for KirBac6.1 (15), are present in *Synechocystis* sp. PCC6803. To gain insights into the physiological roles of these K^+^ channel homologs in response to high ionic stress and high osmolarity, we generated individual mutants in which the entire gene of each K^+^ channel homolog was deleted and examined their growth on BG11 agar medium supplemented with 250 mM KCl, NaCl, or sorbitol (Figs. 1*A* and S1*A*). Under high KCl conditions, Δ*gluR0* grew better than the wildtype and the other K^+^ channel mutants on agar medium. Reintroduction of GluR0 restored the wildtype growth to Δ*gluR0*, confirming that the *gluR0* mutation caused the phenotype (Figs. 1*B* and S1*B*). These data suggested that GluR0 is involved in adaptation to high KCl stress on agar medium. However, no significant difference in growth was observed between the wildtype and Δ*gluR0* in liquid medium supplemented with 250 mM KCl (Fig. S2).

### PM-localized GluR0 confers K^+^-uptake activity

**Figure 2:**
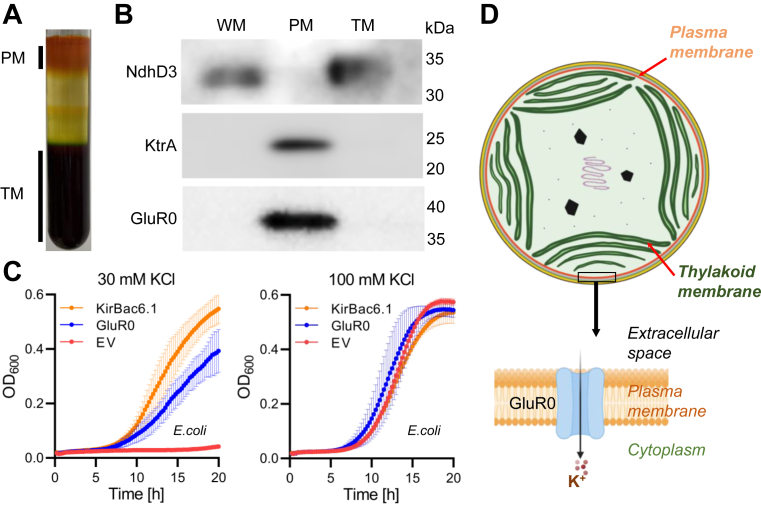
**Plasma membrane (PM) localization and K^+^-uptake activity of GluR0.***A*, membrane fractions were isolated from *Synechocystis* expressing His-tag–fused GluR0 and separated by sucrose density gradient into PM and thylakoid membrane (TM). *B*, immunoblot detection of His-tagged GluR0 in *Synechocystis* using the corresponding antibody. NdhD3 and KtrA were used as marker proteins for TM and PM, respectively. Due to the intrinsic properties of membrane proteins, their apparent molecular mass often deviates from the calculated molecular mass (49, 50). The amounts of total protein loaded onto each lane were 0.2 μg for detection of His-tagged GluR0 and 0.5 μg for detection of KtrA and NdhD3. Data are representative of three biological replicates. *C*, complementation test of a K^+^ uptake transporter-deficient *Escherichia. coli* strain (LB2003) expressing GluR0, KirBac6.1, or containing the empty vector pSTV28, growing in K^+^ minimal medium with 30 mM or 100 mM KCl. Error bars indicate ± SD (n = 5). *D*, subcellular localization of GluR0 in the PM of *Synechocystis*. WM, whole membrane fraction.

To determine the subcellular localization of GluR0, His-tagged GluR0 was generated and expressed in *Synechocystis* (Fig. S1*B*). We prepared both TM-enriched and PM-enriched fractions from the transformed cells by sucrose gradient (Fig. 2*A*) (20, 21). Immunoblot analysis using anti-KtrA antibody (PM marker) and anti-NdhD3 antibody (TM marker) showed that GluR0 was mainly present in the PM-enriched fraction (Fig. 2*B*) (22). KtrA and GluR0 were poorly detected in the whole membrane (WM) fraction because of the low concentration of PM in the WM fraction. Next, to evaluate its transport activity, GluR0 was expressed in the K^+^-uptake activity–defective *E. coli* strain LB2003 (Fig. 2*C*) (23). GluR0 restored the growth of *E. coli* LB2003 in medium containing 30 mM KCl, similar to the positive control, KirBac6.1 (24). In the same medium, the cells containing the empty vector did not grow. These results suggested that GluR0 functions as a K^+^ uptake system in the PM in *Synechocystis* (Fig. 2*D*).

### Membrane orientation of GluR0 is opposite to that of a canonical K^+^ channel

**Figure 3:**
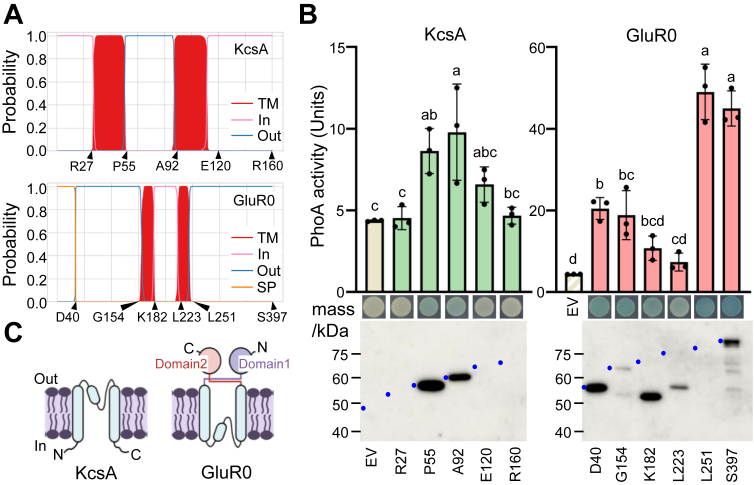
**Analysis of GluR0 membrane topology.***A*, positions of the possible transmembrane domains of KcsA and GluR0 predicted by DeepTMHMM (https://dtu.biolib.com/DeepTMHMM). R27, P55, A92, E120, and R160 of KcsA and D40, G154, K182, L223, and L251 of GluR0 were selected as PhoA fusion sites. *B*, PhoA activity and detection by immunoblotting in *Escherichia coli* UT5600 expressing KcsA-PhoA (*green*) or GluR0-PhoA (*red*) fusion proteins. Different letters indicate significant differences (n = 3; one-way ANOVA/Tukey–Kramer test, *p* < 0.05). The data for the empty vector (EV), shown in *yellow*, are the same in both graphs. For the qualitative assay, cells were grown on solid medium with a chromogenic indicator. On the immunoblot, *blue dots* mark the size of the expected bands for the different PhoA fusion constructs. *C*, membrane topology of KcsA and GluR0. The orientation of GluR0 is opposite to that of the canonical K^+^ channel, KcsA. In, inside the cell; Out, outside the cell; SP, signal peptide; TM, transmembrane.

The tertiary structure of GluR0 has been solved, and it has been described as a glutamate receptor homolog (17). The N- and C-terminal regions of GluR0 are presumed to be localized outside the cell (16, 17). However, since GluR0 contains a canonical K^+^ channel domain similar to KcsA, the N-terminal and C-terminal domains might be located intracellularly instead. To verify which model is correct, we tested the membrane topology of GluR0 by creating alkaline phosphatase (PhoA) fusion proteins of GluR0 and KcsA and determining their location relative to the membrane (25). GluR0 expressed in *E. coli* was functional, indicating it had the correct membrane topology. The PhoA fusion sites were selected adjacent to the predicted transmembrane segments (Fig. 3*A*). When PhoA is translocated to the periplasm, it forms a dimer, creating a stable, enzymatically active structure in the periplasm (26, 27). PhoA fusions at P55 and A92 in KcsA, and at D40, G154, L251, and S397 in GluR0, had relatively high activities, although some values were not statistically significant, indicating a periplasmic location of PhoA (Fig. 3*B*). The fusions at R27, E120, and R160 in KcsA, and K182 and L223 in GluR0 showed relatively low activities, indicating a cytoplasmic location for PhoA (Fig. 3*B*). PhoA fusions at E120 and R160 in KcsA, and at K182 and L223 in GluR0, did not yield statistically significant evidence for cytoplasmic localization. Since the N-terminal hydrophobic sequence of GluR0 might be a signal peptide (17), this region was not included in the topology model. Immunoblotting detected protein bands corresponding to the estimated molecular weight of the fusion proteins that showed relatively high PhoA activity. In contrast, some fusion protein bands were not detected, probably because of protein instability and degradation. The instability of PhoA-fusion proteins has been previously reported (25, 28, 29, 30). In particular, fusion proteins in which the PhoA moiety remains in the cytoplasm may be unstable. Our data indicate that the overall structure of GluR0 is opposite to that of KcsA (Fig. 3*C*).

### Requirement of the soluble domains for GluR0 function

**Figure 4:**
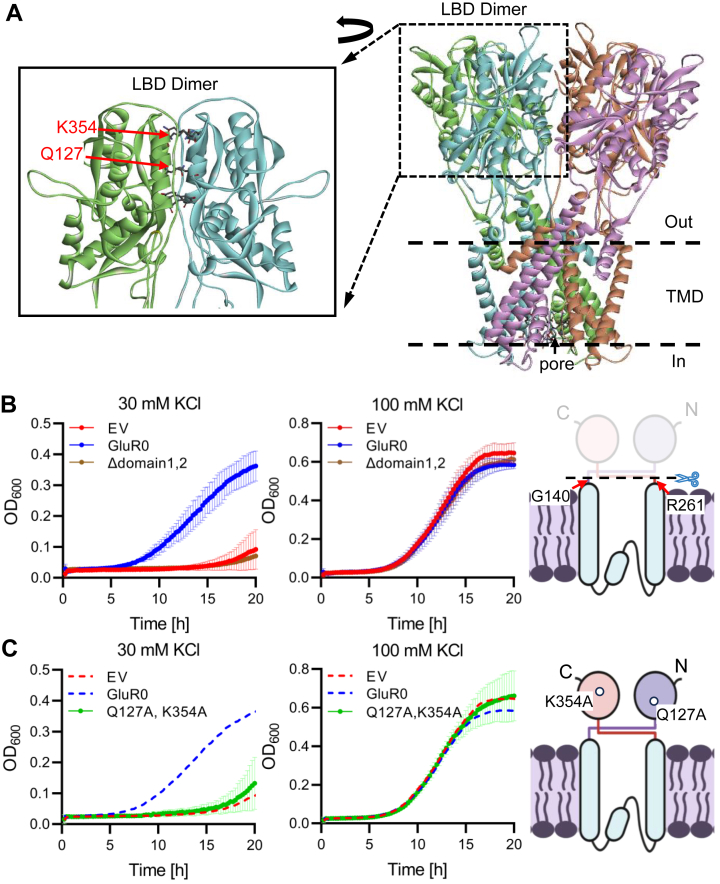
**Importance of soluble domains for GluR0-mediated K^+^-transport activity.***A*, structural model of GluR0. LBD dimer sites (Q127 and K354) are shown on the *left*. *B* and *C*, growth test of *Escherichia coli* LB2003 containing the GluR0-Δdomain1,2 mutant (*B*) or the GluR0-Q127A, K354A mutant (*C*). The growth conditions were the same as in Figure 2*C*. Error bars indicate ±SD (*n* = 3). The growth curves for *E. coli* LB2003 containing pSTV28 (empty vector [EV]) and GluR0 in (*C*) (indicated by the *red dashed line* [EV] and the *blue dashed line* [GluR0]) are the same as those shown in (*B*). The drawings on the *right* depict the structure of the constructs. LBD, ligand-binding domain.

GluR0 is a glutamate receptor that contains the K^+^ selectivity pore of a K^+^ channel sandwiched between the N-terminal (1–140 residues) and C-terminal (256–385 residues) soluble regions (17) (Fig. 4*A*). A construct lacking the N-terminal and C-terminal regions (GluR0Δdomain1,2) failed to restore the growth of the *E. coli* LB2003 mutant in medium containing 30 mM KCl (Fig. 4*B*). These domains have been shown to be essential for the gating mechanism and structural assembly through dimerization (17). Q127 in the N-terminal region and K354 in the C-terminal region are known to be required for dimerization (17) (Fig. 4*A*). When both residues were replaced with Ala (GluR0-Q127A, K354A) the construct failed to restore the growth of LB2003 (Fig. 4*C*). These results suggest that the N-terminal and C-terminal regions of GluR0, along with their inherent dimerization capability, are required for the function of GluR0.

### GluR0 protects against cell shrinkage caused by high concentrations of KCl

**Figure 5:**
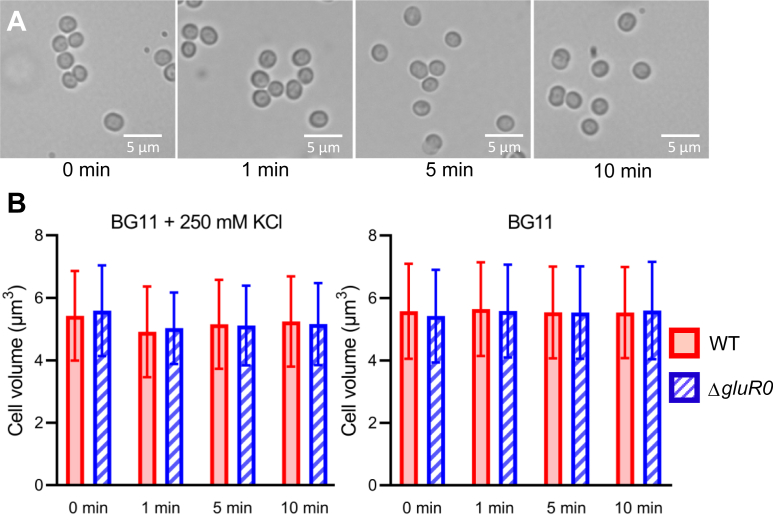
**Cell size change of the *Synechocystis* WT and Δ*gluR0* caused by high KCl.***A*, representative image of *Synechocystis* exposed to BG11 medium with 250 mM KCl for 1 min, 5 min, or 10 min. *B*, determination of cell volume of WT and Δ*gluR0* before and during treatment with BG11 medium with 250 mM KCl. The significance of differences between the WT and Δ*gluR0* was analyzed by Student’s *t* test. Error bars indicate ±SD (*n* = 286–690).

To examine whether GluR0 is involved in cell volume regulation in response to high KCl, we compared the cell volumes of the wildtype and Δ*gluR0* in medium containing 250 mM KCl (Fig. 5). The wildtype and Δ*gluR0* cultured in the normal medium were subjected to KCl upshock, and then the cells were observed under a microscope at 1, 5, and 10 min. There was no difference in the apparent cell volume of the wildtype and Δ*gluR0* during this extended exposure assay.

**Figure 6:**
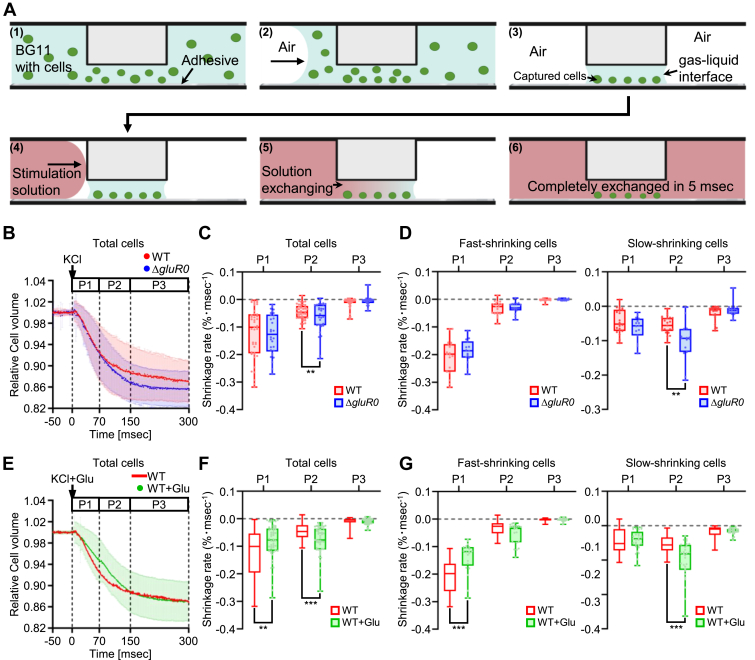
***Synechocystis* cell volume changes in response to KCl upshock treatment with the on-chip instantaneous solution exchange method.***A*, sequence of steps during the rapid exchange of the external solution surrounding *Synechocystis* cells on the microfluidic chip device (1). The cell suspended in the BG11 medium was applied from the inlet, and cells could be trapped by adhesive proteins (Cell-Tak) coated on the *bottom* of the stage (2, 3). Air was introduced into the microchannel to remove the medium outside the narrow stage. Eventually, a gas–liquid interface containing captured cells would form at the stage with a narrow gap (5 μm height) (4, 5, 6). The BG11 medium supplemented with 250 mM KCl was flown to the microchannel to replace the medium of captured cells completely within less than 5 ms and supply a K^+^ upshock. This process was recorded by a high-speed camera at 1000 fps, and the relative cell volume was measured. *B*, relative cell volume–time curve of the WT and Δ*gluR0* (total cells). Cell volume change of cells after treatment with BG11 with 250 mM KCl was calculated. The cell volume was normalized to the initial prestimulus volume. The whole process was divided into three phases: P1, 0 to 70 ms; P2, 70 to 150 ms; and P3, 150 to 300 ms. *C*, shrinkage rate of total cells after 250 mM KCl upshock. *D*, shrinkage rate of fast-shrinking cells (*left*) and slow-shrinking cells (*right*) after 250 mM KCl upshock. *E*, relative cell volume–time curve of the WT after treatment with BG11 with 250 mM KCl and 100 μM glutamate. The curve of the WT treated with only 250 mM KCl (indicated by the *red line*) is the same as that shown in (*B*). *F*, shrinkage rate of total cells after 250 mM KCl and 100 μM glutamate upshock. The shrinkage rate of WT treated with only 250 mM KCl (indicated by an *empty red-lined box*) is the same as that shown in (*C*). *G*, shrinkage rate of fast-shrinking cells (*left*) and slow-shrinking cells (*right*) after 250 mM KCl and 100 μM glutamate upshock. The shrinkage rate of the WT treated with only 250 mM KCl (indicated by an *empty red-lined box*) is the same as that shown in (*C*). The significance of differences was analyzed by Student’s *t* test (∗∗∗*p* < 0.001; ∗∗*p* < 0.01; and ∗*p* < 0.05). Error bars in *B*–*G* indicate ±SD (total cells: WT [*n* = 42], Δ*gluR0* [*n* = 30], WT + Glu [*n* = 52]; fast-shrinking cells: WT [*n* = 19], Δ*gluR0* [*n* = 15], WT + Glu [*n* = 21]; slow-shrinking cells: WT [*n* = 23], Δ*gluR0* [*n* = 15], WT + Glu [*n* = 29]).

To measure transient changes in cell volume in response to rapid replacement of the external solution, a microfluid device, which had been developed to enable extremely fast replacement (μ-sec level) of the culture medium, was used (19). Cell volume measurements were performed using microfluidic chips as described previously (19) (Fig. 6*A*). This process was recorded by a high-speed camera at 1000 fps, and the relative cell volume of the wildtype and the Δ*gluR0* was measured (Fig. 6*B*). We observed that the cellular shrinkage profile consisted of three phases (Fig. 6*B*): phase 1 (P1, 0–70 ms), during which the shrinkage curves of the wildtype and Δ*gluR0* essentially overlapped; phase 2 (P2, 70–150 ms), where the wildtype exhibited a relatively slower shrinkage velocity compared with the Δ*gluR0*; and phase 3 (P3, 150–300 ms), during which the curves of both strains reached a plateau. Upon exposure to a 250 mM KCl upshock, the wildtype exhibited shrinkage rates similar to those of the Δ*gluR0* during P1 and P3. However, the wildtype displayed a significantly lower shrinkage rate in P2 compared with the Δ*gluR0* (Fig. 6*C*). In addition, based on the degree of shrinkage at 70 ms, we divided the total cell population into two groups: a fast-shrinking group (shrinkage levels above the mean) and a slow-shrinking group (shrinkage levels below the mean) (Fig. 6*D*). In the fast-shrinking group, there were no significant differences in shrinkage speeds between the wildtype and Δ*gluR0* across P1, P2, and P3. However, in the slow-shrinking group, the wildtype showed a lower shrinkage rate during P2 compared with the Δ*gluR0*, consistent with the results from the total cell population analysis. With the addition of 100 μM glutamate, the shrinkage rate of the wildtype was reduced in P1 relative to the KCl treatment without glutamate (Fig. 6, *E* and *F*). In P2, glutamate-treated cells shrank faster than cells treated without glutamate. In P3, neither group showed significant differences. A significant reduction in the shrinkage rate during P1 was observed exclusively in the fast-shrinking group, whereas a significant increase in P2 was found only in the slow-shrinking group (Fig. 6*G*). The results suggest that GluR0 helps prevent cell shrinkage during KCl upshock and that this protective effect is further mediated by the addition of glutamate.

## Discussion

This study shows that GluR0 has K^+^ transport activity in *E. coli* and is localized in the PM of *Synechocystis* sp. PCC 6803 (Fig. 2). It is also the first report of a physiological function of GluR0 in cell volume maintenance during high KCl stress (Fig. 6). GluR0 is considered to be an ancestral homolog of GluR animal glutamate receptors (16). The membrane topology of GluR0, as determined through functional expression in *E. coli*, placed both the N- and C-terminal regions outside the cell, which was the opposite of the topology of the KcsA K^+^ channel (Fig. 3). This difference sheds light on how GluR0 may have evolved from K^+^ channels as a molecular adaptation to environmental changes. Nevertheless, since GluR0 possesses two transmembrane domains and the signature Gly-Tyr-Gly sequence of K^+^ channels, its fundamental structure resembles KcsA from *Streptomyces coelicolor* (31). KcsA exhibits K^+^ transport activity in response to both increases and decreases in membrane tension (32). Similar to KcsA, GluR0 might contribute to the response to membrane tension when cells are exposed to a high KCl solution.

The growth of Δ*gluR0* on high KCl exceeded that of the wildtype (Fig. 1). In a culture medium with high concentrations of KCl outside the cell, the absence of GluR0 might contribute to reducing the rate of excessive K^+^ influx into the cell. This growth difference was not observed with NaCl or sorbitol. KCl upshock caused a more gradual rate of cell shrinkage in the wildtype than the Δ*gluR0* (Fig. 6, *B*–*D*). Shabala *et al.* (2) reported that 40% of upregulated genes differed during the response to NaCl and sucrose treatment, indicating that *E. coli* is specifically adapted to respond to ionic *versus* nonionic osmolytes. Changes in either extracellular ion strength or osmotic pressure result in K^+^ flux across the membrane (8, 18).

*Synechocystis* has two major K^+^ transport systems, KtrABE, which is a Na^+^-dependent K^+^ transporter, and Kdp, which is a K^+^ pump (6). Adaptation to osmotic upshock because of KtrABE activity is relatively slow (more than 500 s). Expression of *kdp* is suppressed under K^+^ sufficient conditions. These transporters are involved in maintaining cellular homeostasis over a relatively long period. In contrast, K^+^ channel homologs may be involved in short-term responses in *Synechocystis*, since no significant phenotypes had been reported for K^+^ channel mutants. Consistent with this, when we examined the growth of our deletion mutants, including Δ*synCaK*, Δ*synK*, and Δ*kirbac6.1*, in medium containing high osmotic pressure and high salt concentration, no changes were observed compared with the wildtype (Fig. 1*A*). During NaCl and sorbitol stress, the growth of Δ*gluR0* was also the same as that of the wildtype. A phenotype was observed for Δ*gluR0* under high KCl concentration conditions. Our results indicate that the wildtype exhibits higher potassium uptake efficiency than Δ*gluR0* (Fig. 6). Under long-term exposure to high KCl concentrations, this relatively high potassium absorption mediated by GluR0 likely results in the slightly compromised growth of the wildtype compared with Δ*gluR0* (Figs. 1 and 6). GluR0 likely functioned as a passive K influx system. These findings underline the challenge of detecting the phenotype of K^+^ channel mutants in *Synechocystis*.

When exposed to hyperosmotic stress, bacteria initially take up K^+^ to counteract the increase in external osmotic pressure and simultaneously synthesize glutamate to compensate for the resulting positive charge (2, 33). However, prolonged accumulation of potassium glutamate has a detrimental effect on cytosolic ionic homeostasis. Consequently, bacteria subsequently shift to the use of nonionic compatible solutes and actively efflux the accumulated potassium glutamate (34, 35), a process that is thought to be mediated, at least in part, by the transport system (36, 37). In *Synechocystis*, the adaptive response might lead to the activation of GluR0 upon binding for effluxed glutamate. Because GluR0 possesses a K^+^-selective filter, its activation is unlikely to have a major physiological impact under conditions of high NaCl or hyperosmotic stress induced solely by sorbitol. Exposure to high external KCl may drive a substantial K^+^ influx through activated GluR0, resulting in intracellular K^+^ overload and, ultimately, growth inhibition (Fig. 1). The absence of the growth difference in liquid medium might be attributed to the fact that the concentration of released glutamate was insufficient to reach the threshold required to activate GluR0 because of rapid dilution by the medium and lower cell density compared with that on agar plates.

Glutamate, in conjunction with its receptors, has been widely reported to participate in neural electrical signaling in animals (38, 39) and long-distance Ca^2+^ signaling in plants (40, 41). Recent studies showed that electrical signaling mechanisms exist in bacterial communities. Specifically, potassium ion channels are closely associated with these processes, including biofilm formation (42, 43, 44). While glutamate in bacteria is involved in acid tolerance (45) and nitrogen assimilation (46, 47), GluR0 may play a role in glutamate-dependent intercellular communication, given its potential glutamate-mediated potassium transport activity.

This study presents a functional characterization of *Synechocystis* GluR0, an ancestral glutamate receptor, as a K^+^ channel with a membrane orientation that is opposite of that of a canonical K^+^ channel in the PM (Fig. 3). GluR0 was shown to be localized in the PM of cyanobacteria where it is involved in the response to environmental changes (Figs. 1, 2 and 6). These characteristics provide GluR0 with the dual capacity to conduct rapid ion transport as a K^+^ channel while sensing the extracellular environment through its ligand-binding domain. This functional synergy likely laid the foundation for the rapid signaling mechanisms observed in its eukaryotic homologs. *Synechocystis* GluR0 is likely a key molecule for understanding K^+^ channels and glutamate receptors in both prokaryotic and eukaryotic cells.

## Experimental procedures

### Growth conditions of *Synechocystis*

For *Synechocystis*, cells were incubated in BG11 medium at 28 °C, supplemented with 20 mM *N*-Tris(hydroxymethyl)methyl-2-aminoethanesulfonic acid–KOH. If necessary, 20 μg/ml of chloramphenicol or kanamycin was added for selection of cells. The cell cultures were maintained in a BR-180LF incubator (TAITEC) or an LPH-411PFDT incubator (Nippon Medical and Chemical Instruments), with light irradiance of 50 μmol/m^2^s supplied with white LEDs.

### Plasmid construction

The primers used for plasmid construction are listed in Table S1. DNA sequences encoding GluR0 and KirBac6.1 were PCR amplified using *Synechocystis* genomic DNA as a template and cloned into the BamHI/SalI sites of pSTV28 (TaKaRa) to generate pSTV28-GluR0 and pSTV28-KirBac6.1, respectively. To generate the GluR0 variants, PCR-based mutations in *gluR0* of pSTV28-GluR0 were performed using corresponding primers. Amplified genes encoding point mutants were inserted into the *Bam*HI/*Sal*I site of pSTV28. DNA cloning was performed using the In-fusion Kit (TaKaRa).

To construct plasmids for disruption of *synCaK*, *sll0536, synK*, *gluR0*, and *kirBac6.1* in the *Synechocystis* genome, 500 bp upstream of each gene were inserted into the *Eco*RV site of pUV19-km, whereas 500 bp downstream were inserted into the *Nco*I site of pUV19-km, respectively. For complementation of Δ*gluR0*, the coding regions of *gluR0*, including 500 bp of upstream sequence, were cloned into the *Aat*II and *Hpa*I sites of pCmptrc (21).

To express His-tag–fused GluR0 in the wildtype *Synechcystis*, the PCR-amplified *gluR0* gene fragment was subcloned into the *Nde*I and *Hpa*I sites of pTCPH2031. By the introduction of the resultant plasmid into the wildtype, the sequence encoding His-tag–fused GluR0 was integrated into a neutral site between *slr2030* and *slr2031* (48). Segregation was completed in BG11 medium with chloramphenicol, followed by confirmation of correct integration by PCR with appropriate primers.

For the plasmid construction for fusions of KcsA-PhoA (pPAB404-KcsA-PhoA) and GluR0-PhoA (pPAB404-GluR0-PhoA), the DNA fragments were amplified by PCR using either pQE82-KcsA (kindly provided by Masayuki Iwamoto and Shigetoshi Oiki) or pPAB404-GluR0 as a template and cloned into the *Bam*HI site of pPAB404 (25).

### Growth and assay conditions of *E. coli*

*E. coli* LB2003 was grown in LB medium (0.5% yeast extract, 1% peptone, and 0.5% KCl) at 30 °C overnight. Cells were harvested by centrifugation and washed and resuspended in minimal medium containing 46 mM Na_2_HPO_4_, 23 mM NaH_2_PO_4_, and 80 mM (NH_4_)_2_SO_4_. The cell suspension was adjusted to an absorbance of 2 at 600 nm and inoculated into fresh K^+^ minimal medium supplemented with 20% glucose, 6 μM FeSO_4_, 0.4 mM MgSO_4_, 25 μg/ml chloramphenicol, 100 μM IPTG, and 30 mM or 100 mM KCl. The cell suspension was incubated in a microplate reader (SYNERGY H1; BioTek) at 30 °C, and absorbance at 600 nm was measured every 15 min for 20 h.

### Fractionation and detection of PMs and TMs

Isolation of PMs and TMs was performed as described previously with slight modifications (20, 21). Briefly, cells were incubated in 1 l of BG11 medium until an absorbance of 1.0 at 730 nm. Cells were harvested and treated with lysozyme. Then, cells were disrupted in a French press three times at 160 MPa, followed by treatment with DNaseI and PMSF. After the centrifugation, the supernatant was collected carefully. To obtain the WM fraction, 1.75 ml of the cell lysate was mixed with 1.75 ml of 10 mM *N*-Tris(hydroxymethyl)methyl-2-aminoethanesulfonic acid–NaOH buffer containing 10 mM NaCl and then ultracentrifuged at 103,000*g* for 30 min at 4 °C. The supernatant was removed, and the pellet corresponding to the WM fraction was collected. To separate PM and TM fractions, 3 ml of the cell lysate was mixed with 2.25 ml of an 80% sucrose solution, and 5 ml of the mixture was added to the bottom of a polypropylene tube. Sucrose solutions (1.5 ml of 42% sucrose, 2.5 ml of 30% sucrose, and 1 ml of 10% sucrose) were layered sequentially above the cell lysate to create a sucrose gradient. Immunoblotting was performed as described (21).

### Membrane topology analysis

Determination of PhoA activity and immunoblotting were performed essentially as previously reported (28). Briefly, for the qualitative assay, *E. coli* UT5600 containing pPAB404-KcsA-PhoA and pPAB404-GluR0-PhoA were grown on LB medium with 50 μg/ml ampicillin overnight at 30 °C. Cells (5 μl of an absorbance of 0.5 at 600 nm) were spotted on LB agar containing 50 μg/ml ampicillin, 0.1 mM IPTG, and 40 μg/ml 5-bromo-4-chloro-3-indoylphosphate and then incubated at 30 °C for 24 h. For the quantitative assay, a midlog phase LB-grown cell culture was supplemented with 0.1 mM IPTG and further incubated for 2 h at 30 °C to induce PhoA fusion protein expression. Cells were collected by centrifugation, and PhoA activity was determined as described (28). For immunoblotting of PhoA fusion proteins, 1 μg of the membrane fraction from an IPTG-induced culture was separated on an SDS-polyacrylamide gel (10%), and proteins were detected using antibodies against PhoA (Rockland) and goat anti-rabbit horseradish peroxidase–conjugated IgG (Merck kGaA).

### Microscopy

A microscope (IX83; Evident) equipped with a spinning disk confocal unit CSU-W1 (Yokogawa Electric Corporation) and CMOS cameras (ORCA-Flash 4.0; Hamamatsu Photonics) was used to image the wildtype and Δ*gluR0* from *Synechocystis* cultures grown to an absorbance of 3.0 at 730 nm. The cell suspension (1 ml) was added to the bottom dish, followed by the addition of 2 M KCl solution to a final concentration of 250 mM KCl. Cell sizes were determined using ImageJ∗ (National Institutes of Health).

### Microfluidic measurement

Microfluidic chips used in this research were prepared as described previously (19). Instantaneous extracellular solution exchange was conducted as described with minor modifications (19). In brief, the microfluidic chip coated with Cell-Tak in advance was mounted onto a microscope (IX73; Olympus Co Ltd). Inlet tubes that introduced cell suspension, air, or the stimulation solution were fixed to the microfluidic chip with tubing sorts. The cell suspension and air inlet were opened first to introduce the cells and create a liquid bridge at the micropillar. After cells were captured at the micropillar, the cell suspension inlet was sealed, and only the liquid bridge remained, whereas the medium around the pillar was expelled by air. Then, the solution stimulation inlet was opened, and the formed liquid bridge was exchanged with the stimulation solution (BG11 medium with only 250 mM KCl or BG11 medium with 250 mM KCl and 100 μM glutamate). This process was observed under the microscope and recorded by a high-speed camera (Fastcam Mini AX50; Photron Ltd) with exposure time and frame rate set at 0.5 ms and 1000 fps. The size of single cells trapped at the pillar was evaluated using MATLAB 2023b. The cell volume of cyanobacteria was calculated by converting the area obtained with ImageJ to volume, assuming that the cells are spherical.

## Supporting information

This article contains supporting information.

## Conflict of interest

The authors declare that they have no conflicts of interest with the contents of this article.

## References

[1] Kempf B., Bremer E. (1998). Uptake and synthesis of compatible solutes as microbial stress responses to high-osmolality environments. Arch. Microbiol..

[2] Shabala L., Bowman J., Brown J., Ross T., McMeekin T., Shabala S. (2009). Ion transport and osmotic adjustment in *Escherichia coli* in response to ionic and non-ionic osmotica. Environ. Microbiol..

[3] Treptow W., Tarek M., Klein M.L. (2009). Initial response of the potassium channel voltage sensor to a transmembrane potential. J. Am. Chem. Soc..

[4] Hagemann M. (2011). Molecular biology of cyanobacterial salt acclimation. FEMS Microbiol. Rev..

[5] Kaneko T., Sato S., Kotani H., Tanaka A., Asamizu E., Nakamura Y. (1996). Sequence analysis of the genome of the unicellular cyanobacterium *Synechocystis* sp. strain PCC6803. II. Sequence determination of the entire genome and assignment of potential protein-coding regions. DNA Res..

[6] Nanatani K., Shijuku T., Takano Y., Zulkifli L., Yamazaki T., Tominaga A. (2015). Comparative analysis of *kdp* and *ktr* mutants reveals distinct roles of the potassium transporters in the model cyanobacterium *Synechocystis* sp. strain PCC 6803. J. Bacteriol..

[7] Zulkifli L., Akai M., Yoshikawa A., Shimojima M., Ohta H., Guy H.R. (2010). The KtrA and KtrE subunits are required for Na^+^-dependent K^+^ uptake by KtrB across the plasma membrane in *Synechocystis* sp. strain PCC 6803. J. Bacteriol..

[8] Matsuda N., Kobayashi H., Katoh H., Ogawa T., Futatsugi L., Nakamura T. (2004). Na^+^-dependent K^+^ uptake ktr system from the cyanobacterium *Synechocystis* sp. PCC 6803 and its role in the early phases of cell adaptation to hyperosmotic shock. J. Biol. Chem..

[9] Berry S., Esper B., Karandashova I., Teuber M., Elanskaya I., Rogner M. (2003). Potassium uptake in the unicellular cyanobacterium *Synechocystis* sp. strain PCC 6803 mainly depends on a Ktr-like system encoded by *slr1509* (*ntpJ*). FEBS Lett..

[10] Lundback A.K., Muller S.A., Engel A., Hebert H. (2009). Assembly of Kch, a putative potassium channel from *Escherichia* coli. J. Struct. Biol..

[11] Checchetto V., Formentin E., Carraretto L., Segalla A., Giacometti G.M., Szabo I. (2013). Functional characterization and determination of the physiological role of a calcium-dependent potassium channel from Cyanobacteria. Plant Physiol..

[12] Checchetto V., Segalla A., Allorent G., La Rocca N., Leanza L., Giacometti G.M. (2012). Thylakoid potassium channel is required for efficient photosynthesis in Cyanobacteria. Proc. Natl. Acad. Sci. U. S. A..

[13] Zanetti M., Teardo E., La Rocca N., Zulkifli L., Checchetto V., Shijuku T. (2010). A novel potassium channel in photosynthetic Cyanobacteria. PLoS One.

[14] Checchetto V., Segalla A., Sato Y., Bergantino E., Szabo I., Uozumi N. (2016). Involvement of potassium transport systems in the response of *Synechocystis* PCC 6803 Cyanobacteria to external pH change, high-intensity light stress and heavy metal stress. Plant Cell Physiol..

[15] Paynter J.J., Andres-Enguix I., Fowler P.W., Tottey S., Cheng W., Enkvetchakul D. (2010). Functional complementation and genetic deletion studies of KirBac channels: activatory mutations highlight gating-sensitive domains. J. Biol. Chem..

[16] Chen G.Q., Cui C., Mayer M.L., Gouaux E. (1999). Functional characterization of a potassium-selective prokaryotic glutamate receptor. Nature.

[17] Mayer M.L., Olson R., Gouaux E. (2001). Mechanisms for ligand binding to GluR0 ion channels: crystal structures of the glutamate and serine complexes and a closed Apo state. J. Mol. Biol..

[18] Nanatani K., Shijuku T., Akai M., Yukutake Y., Yasui M., Hamamoto S. (2013). Characterization of the role of a mechanosensitive channel in osmotic down shock adaptation in *Synechocystis* sp PCC 6803. Channels (Austin).

[19] Kaneko S., Hirotaka S., Tsujii M., Maruyama H., Uozumi N., Arai F. (2024). Instantaneous extracellular solution exchange for concurrent evaluation of membrane permeability of single cells. Lab Chip.

[20] Omata T., Murata N. (1985). Electron-transport reactions in cytoplasmic and thylakoid membranes prepared from the Cyanobacteria (blue-green algae) *Anacystis Nidulans* and *Synechocystis* PCC 6714. Biochim. Biophys. Acta.

[21] Tsujii M., Kobayashi A., Kano A., Kera K., Takagi T., Nagata N. (2024). Na^+^-driven pH regulation by Na^+^/H^+^ antiporters promotes photosynthetic efficiency in Cyanobacteria. Plant Physiol..

[22] Zhang P.P., Battchikova N., Jansen T., Appel J., Ogawa T., Aro E.M. (2004). Expression and functional roles of the two distinct NDH-1 complexes and the carbon acquisition complex NdhD3/NdhF3/CupA/Sll1735 in sp PCC 6803. Plant Cell.

[23] Schlosser A., Meldorf M., Stumpe S., Bakker E.P., Epstein W. (1995). TrkH and its homolog, TrkG, determine the specificity and kinetics of cation transport by the Trk system of *Escherichia coli*. J. Bacteriol..

[24] Zubcevic L., Bavro V.N., Muniz J.R., Schmidt M.R., Wang S., De Zorzi R. (2014). Control of KirBac3.1 potassium channel gating at the interface between cytoplasmic domains. J. Biol. Chem..

[25] Uozumi N., Nakamura T., Schroeder J.I., Muto S. (1998). Determination of transmembrane topology of an inward-rectifying potassium channel from *Arabidopsis thaliana* based on functional expression in *Escherichia coli*. Proc. Natl. Acad. Sci. U. S. A..

[26] Boyd D., Manoil C., Beckwith J. (1987). Determinants of membrane protein topology. Proc. Natl. Acad. Sci. U. S. A..

[27] Brickman E., Beckwith J. (1975). Analysis of the regulation of *Escherichia coli* alkaline phosphatase synthesis using deletions and *phi80* transducing phages. J. Mol. Biol..

[28] Sato Y., Nanatani K., Hamamoto S., Shimizu M., Takahashi M., Tabuchi-Kobayashi M. (2014). Defining membrane spanning domains and crucial membrane-localized acidic amino acid residues for K^+^ transport of a Kup/HAK/KT-type *Escherichia coli* potassium transporter. J. Biochem..

[29] Zimmann P., Puppe W., Altendorf K. (1995). Membrane topology analysis of the sensor kinase KdpD of *Escherichia coli*. J. Biol. Chem..

[30] Kato S., Mihara H., Kurihara T., Takahashi Y., Tokumoto U., Yoshimura T. (2002). Cys-328 of IscS and Cys-63 of IscU are the sites of disulfide bridge formation in a covalently bound IscS/IscU complex: implications for the mechanism of iron-sulfur cluster assembly. Proc. Natl. Acad. Sci. U. S. A..

[31] Doyle D.A., Morais Cabral J., Pfuetzner R.A., Kuo A., Gulbis J.M., Cohen S.L. (1998). The structure of the potassium channel: molecular basis of K^+^ conduction and selectivity. Science.

[32] Iwamoto M., Oiki S. (2021). Hysteresis of a tension-sensitive K^+^ channel revealed by time-lapse tension measurements. JACS Au..

[33] Csonka L.N. (1989). Physiological and genetic responses of bacteria to osmotic stress. Microbiol. Rev..

[34] Cayley S., Lewis B.A., Guttman H.J., Record M.T. (1991). Characterization of the cytoplasm of *Escherichia coli* K-12 as a function of external osmolarity. Implications for protein-DNA interactions in vivo. J. Mol. Biol..

[35] Goude R., Renaud S., Bonnassie S., Bernard T., Blanco C. (2004). Glutamine, glutamate, and alpha-glucosylglycerate are the major osmotic solutes accumulated by *Erwinia chrysanthemi* strain 3937. Appl. Environ. Microbiol..

[36] Becker M., Borngen K., Nomura T., Battle A.R., Marin K., Martinac B. (2013). Glutamate efflux mediated by *Corynebacterium glutamicum* MscCG, *Escherichia coli* MscS, and their derivatives. Biochim. Biophys. Acta.

[37] Kawasaki H., Martinac B. (2020). Mechanosensitive channels of *Corynebacterium glutamicum* functioning as exporters of L-glutamate and other valuable metabolites. Curr. Opin. Chem. Biol..

[38] Levite M. (2017). Glutamate, T cells and multiple sclerosis. J. Neural Transm. (Vienna).

[39] Reiner A., Levitz J. (2018). Glutamatergic signaling in the central nervous system: ionotropic and metabotropic receptors in concert. Neuron.

[40] Qiu X.M., Sun Y.Y., Ye X.Y., Li Z.G. (2019). Signaling role of glutamate in plants. Front Plant Sci..

[41] Yu B., Liu N., Tang S., Qin T., Huang J. (2022). Roles of glutamate receptor-like channels (GLRs) in plant growth and response to environmental stimuli. Plants (Basel).

[42] Liu J., Prindle A., Humphries J., Gabalda-Sagarra M., Asally M., Lee D.Y. (2015). Metabolic co-dependence gives rise to collective oscillations within biofilms. Nature.

[43] Prindle A., Liu J., Asally M., Ly S., Garcia-Ojalvo J., Suel G.M. (2015). Ion channels enable electrical communication in bacterial communities. Nature.

[44] Humphries J., Xiong L., Liu J., Prindle A., Yuan F., Arjes H.A. (2017). Species-independent attraction to biofilms through electrical signaling. Cell.

[45] Feehily C., Karatzas K.A. (2013). Role of glutamate metabolism in bacterial responses towards acid and other stresses. J. Appl. Microbiol..

[46] Okuhara H., Matsumura T., Fujita Y., Hase T. (1999). Cloning and inactivation of genes encoding ferredoxin- and NADH-dependent glutamate synthases in the cyanobacterium *plectonema boryanum*. Imbalances in nitrogen and carbon assimilations caused by deficiency of the ferredoxin-dependent enzyme. Plant Physiol..

[47] Herrero A., Muro-Pastor A.M., Flores E. (2001). Nitrogen control in Cyanobacteria. J. Bacteriol..

[48] Kamei A., Ogawa T., Ikeuchi M. (1998). Identification of a novel gene (*slr2031*) involved in high-light resistance in the cyanobacterium *Synechocystis* sp. PCC 6803. Photosynthesis: Mechanisms and Effects.

[49] Rath A., Glibowicka M., Nadeau V.G., Chen G., Deber C.M. (2009). Detergent binding explains anomalous SDS-PAGE migration of membrane proteins. Proc. Natl. Acad. Sci. U. S. A..

[50] Zhang P., Battchikova N., Jansen T., Appel J., Ogawa T., Aro E.M. (2004). Expression and functional roles of the two distinct NDH-1 complexes and the carbon acquisition complex NdhD3/NdhF3/CupA/Sll1735 in *Synechocystis* sp PCC 6803. Plant Cell.

