# Supplementary material for: An ancestral glutamate receptor mediates cell volume regulation during high K+ stress in cyanobacteria
Source: J Biol Chem. 2026 Mar 21;302(5):111396. doi: 10.1016/j.jbc.2026.111396 (PMC13099474; doi:10.1016/j.jbc.2026.111396)
Supplement: Supporting Figures and Table [file mmc1.pdf]

Supplemental Table S1.

Supplemental Table S1. Primers used in this study

Name	Sequence (5'to 3')
**SynCaK_U600_Fw1**	TAGAGGATCCCCGATACTAGCTTTGGCGGTGAG
**SynCaK_U600_Rv1**	AGATCTGAATTCGATCCTAAAACCTCACTTAAAACAGG
**SynCaK_D600_Fw1**	ATCTGAATTCCCATGTATAAGGAATACCGTCATTATGC
**SynCaK_D600_Rv1**	CTCGGTACCCCCATGGTGATTAGATTTCCCGC
**Sll0536-U500_Fw1**	TAGAGGATCCCCGATTCGCCACGGAGGCCCAC
**Sll0536-U500_Rv1**	AGATCTGAATTCGATAGTTAAACGGACTAAACGGCGATCGGC
**Sll0536-D500_Fw1**	ATCTGAATTCCCATGCCTCCATCGATACACTCTGG
**Sll0536-D500_Rv1**	CTCGGTACCCCCATGATGCACCACCGTTGACC
**SynK-U500_Fw1**	TAGAGGATCCCCGATGGGGAGGACTGGCTACTAG
**SynK-U500_Rv1**	AGATCTGAATTCGATGGTAAAAAATAGTAATAAAACAATTGACCAAAAC
**SynK-D500_Fw1**	ATCTGAATTCCCATGAGCCCATGCGAGAAAATTC
**SynK-D500_Rv1**	CTCGGTACCCCCATGCCCAACAGCAGGAACGC
**GluR0-U500_Fw1**	TAGAGGATCCCCGATCAACTTTTCCCGATGGCAC
**GluR0-U500_Rv1**	AGATCTGAATTCGATTTGCCAGACAGAGCCTC
**GluR0-D500_Fw1**	ATCTGAATTCCCATGACCCTGGTAAAACTTATAATACCTTCTG
**GluR0-D500_Rv1**	CTCGGTACCCCCATGCTGCCGTGGGCACATTTTTTG
**KirBac6.1-U500_Fw1**	TAGAGGATCCCCGATGGCTATTGCCCCTAGCGG
**KirBac6.1-U500_Rv1**	AGATCTGAATTCGATCAAGACTTTTCAGCCATCATTGC
**KirBac6.1-D500_Fw1**	ATCTGAATTCCCATGATCTACTTTTAGTTTATTTGGCAATGAGTTC
**KirBac6.1-D500_Rv1**	CTCGGTACCCCCATGAGCTTGGTCTATAACGCAACC
**pGluR0-GluR0-F**	GCTTCGTTAATACAGACGTCCAACTTTTCCCGATGGCAC
**pGluR0-GluR0-R**	ATCCAATGTGAGGTTAACTTAGCTAGGGCTTTCTCC
**pKtrEGluR0hisF2**	TGGTTATCTGCCATATGTTAATCCTGCGTCGCCTAATAC
**pKtrEGluR0hisR**	ATCCAATGTGAGGTTAACTTAATGATGATGATGATGATGG
**GluR0-BamHI-start-F**	GAGCTCGGTACCCGGGGATCCATGTTAATCCTGCGTCGCCTA
**GluR0-D40-BamHI-R**	CTGCAGGTCGACTCTAGAGGATCCGTCTACTGTCTCCACCCC
**GluR0-G154-BamHI-R**	CTGCAGGTCGACTCTAGAGGATCCGCCAAAAAAAGGCGAAAATC
**GluR0-K182-BamHI-R**	CTGCAGGTCGACTCTAGAGGATCCTTTGCGATGTTCTGCTAA
**GluR0-L223-BamHI-R**	CTGCAGGTCGACTCTAGAGGATCCCAGTTGCCCTAACTTAGT
**GluR0-L251-BamHI-R**	CTGCAGGTCGACTCTAGAGGATCCAAGAGCCGTGGAAAAAGC
**GluR0-S397-BamHI-R**	CTGCAGGTCGACTCTAGAGGATCCGCTAGGGCTTTCTCCTAT
**GluR0_seq_Fw1**	CATAGAGGGCATTACCTTCA
**KcsA-BamHI-start-F**	GCTCGGTACCCGGGGATCCATGCCACCCATGCTGTCC
**KcsA-R27-BamHI-R**	GGTCGACTCTAGAGGATCCCCTCCAGTGCAGCGCACT
**KcsA-P55-BamHI-R**	GGTCGACTCTAGAGGATCCCGGTGCGCCGCGCTCAGC
**KcsA-A92-BamHI-R**	GGTCGACTCTAGAGGATCCGGCCACGAGCCGGCCCCA
**KcsA-R120-BamHI-R**	GGTCGACTCTAGAGGATCCCTCTTGTTCCCGGCCGAC
**KcsA-R160-BamHI-R**	GGTCGACTCTAGAGGATCCCCGGCGGTTGTCGTCGAG
**pSTV28-BamHI-GluR0-F**	TCGGTACCCGGGGATCCATGTTAATCCTGCGTCGCCTAATAC
**pSTV28-SalI-GluR0-R**	CATGCCTGCAGGTCGACTTAGCTAGGGCTTTCTCCTATGTTTTG
**GluR0_335bp_colonyPCR_R**	CTAATGGGACCAATTAAAATATCC
**pSTV28-BamHI-GluR0-G140-R261-F**	TCGGTACCCGGGGATCCATGGGCAAACCGGTGAGTTTATG
**pSTV28-SalI-GluR0-G140-R261-R**	CATGCCTGCAGGTCGACTTATCTAAACAAAGGTGTTGCACTAGCTTC
**GluR0-Q127A-F**	CACCGCACCCTATTTCAGTAG
**GluR0-Q127A-R**	CTACTGAAATAGGGTGCGGTG
**GluR0-K354A-F**	CCTTTGCAAGCAACTATCAATGTG
**GluR0-K354A-R**	CACATTGATAGTTGCTTGCAAAGG
**SynCaK_geno_Fw1**	CAACAGGGGAATAGGTCACG
**SynCaK_geno_Rv1**	CCAGCAGTAACCAGTAGGTC
**slr2031ORF-163bp_F**	CCATGGGGAAGTTTGCTGG
**slr2031_316bp_R**	CTTGGCGCAATTGACGGTC

Supplementary Figure S1

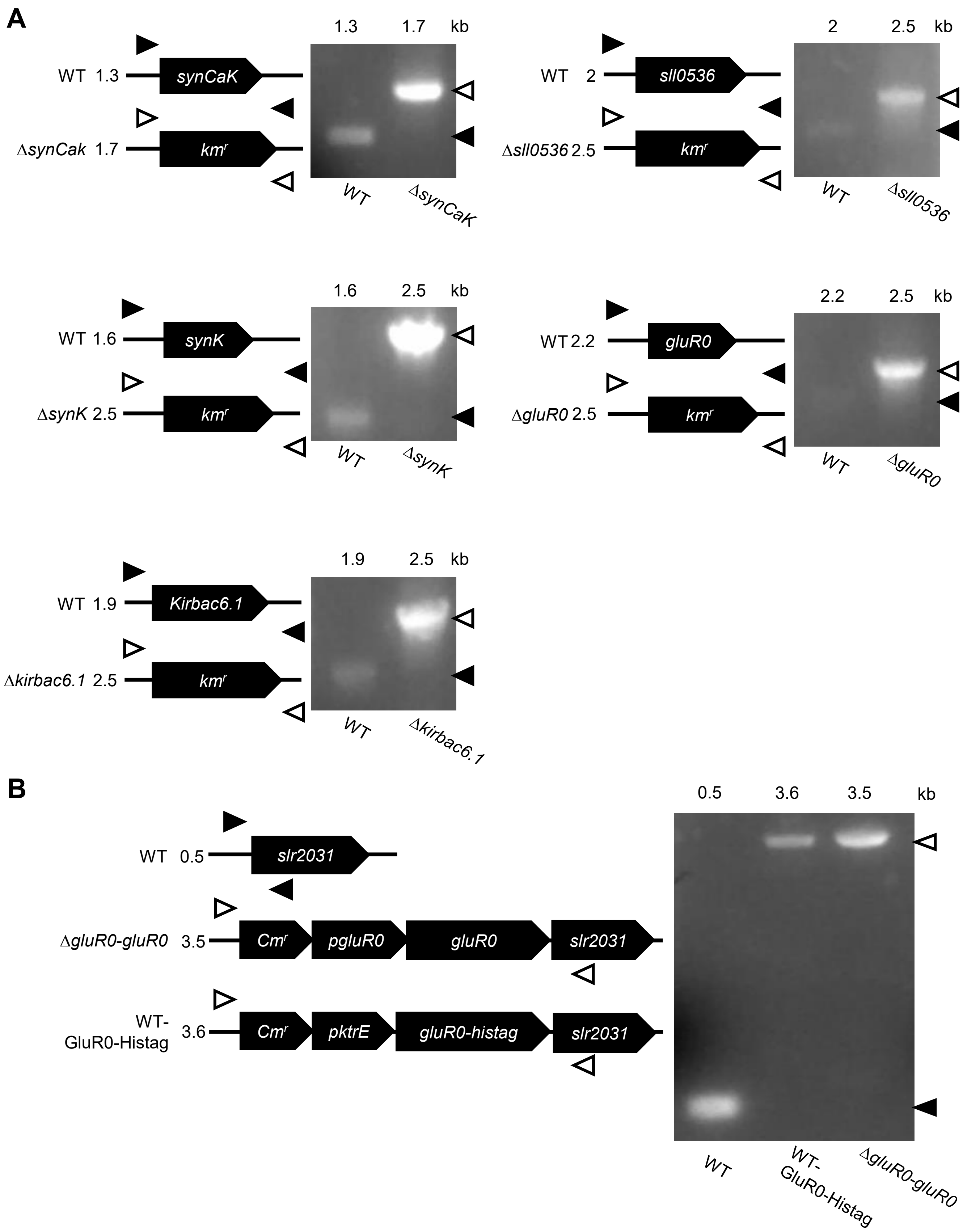

Supplemental Figure S1. Generation of disruption mutants, complemented strains and introduction of His-tagged GluR0.

Supplementary Figure S2

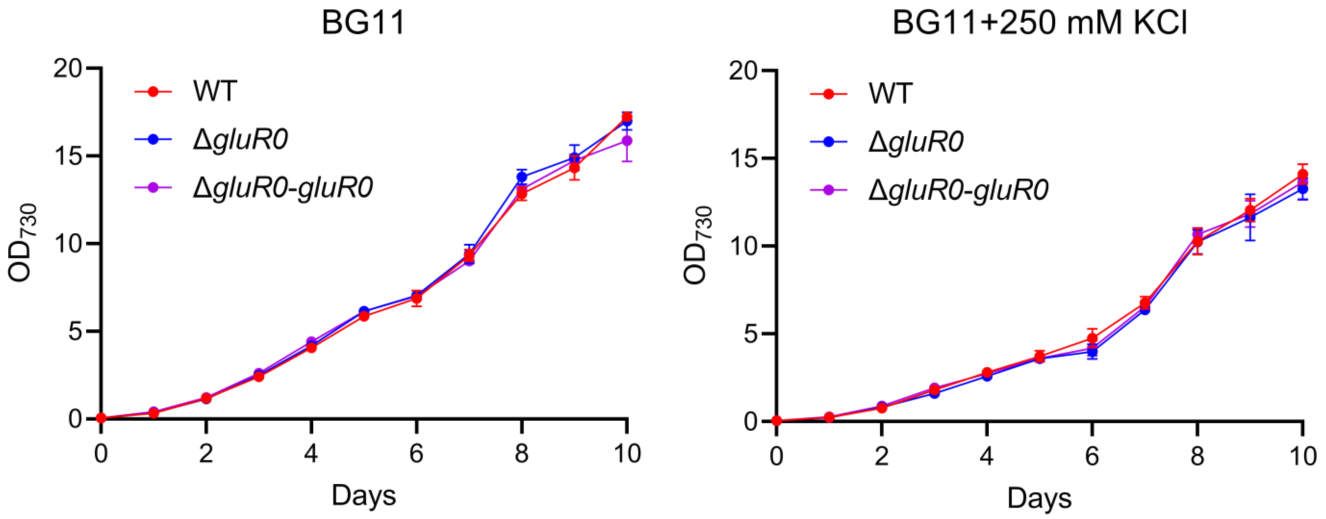

Supplemental Figure S2. Growth of the *Synechocystis* wild type and *gluR0* mutant in liquid BG11 medium or BG11 medium supplied with 250 mM KCl. Cells were incubated from $OD_{730} = 0.05$ at 28˚ C with light irradiance of 50 µmol/m^{2}s supplied with white LEDs and OD_{730} was measured everyday. Error bars indicate ± SD (n = 3).
